# Italian Adaptation of the No-Mobile-Phone-Phobia Questionnaire: Factorial Validity with the ESEM Technique and Population-Based Cut-Off Scores

**DOI:** 10.3390/ejihpe15080166

**Published:** 2025-08-21

**Authors:** Sergio Traficante, Luigi Tinella, Antonella Lopez, Sergio A. Useche, Sjaan Koppel, Giuseppina Spano, Elisabetta Ricciardi, Rosa Napoletano, Andrea Bosco, Alessandro O. Caffò

**Affiliations:** 1 Department of Educational Sciences, Psychology, Communication, University of Bari, 70121 Bari, Italy; sergio.traficante@uniba.it (S.T.); rosa.napoletano@uniba.it (R.N.); andrea.bosco@uniba.it (A.B.); alessandro.caffo@uniba.it (A.O.C.); 2 Department of Humanities, Philosophy and Education, University of Salerno, 84084 Salerno, Italy; ltinella@unisa.it; 3 Department of Humanities, Social Science, and Education, University of Molise, 86100 Campobasso, Italy; 4 Faculty of Psychology, University of Valencia, 46010 Valencia, Spain; sergio.useche@uv.es; 5 Monash University Accident Research Centre (MUARC), Monash University, Melbourne 3800, Australia; sjaanie.koppel@monash.edu; 6 Department of Psychology and Health Science, Pegaso Telematic University, 80132 Naples, Italy; giuseppina.spano@unipegaso.it; 7 Department of Precision and Regenerative Medicine and Ionian Area, University of Bari, 70121 Bari, Italy; elisabetta.ricciardi@uniba.it

**Keywords:** nomophobia, exploratory structural equation modeling, four-factor configuration, cut-off scores, NMP-Q adaptation

## Abstract

Nomophobia is a multifaceted phenomenon characterized by fear and anxiety when individuals feel disconnected from their technological environment. Its assessment remains difficult due to limited tools and lack of empirically supported cut-off points. This study aimed to contribute to the Italian validation of the Nomophobia Questionnaire (NMP-Q), testing a four-factor structure and establishing normative data by age and gender. Data were collected from 1447 participants. Exploratory Structural Equation Modeling (ESEM) assessed different factorial configurations. A bifactor ESEM (B-ESEM) with a four-factor solution showed the best fit (CFI = 0.95; TLI = 0.94; RMSEA = 0.06), offering a more accurate representation than the three-factor model. Scores were computed for the 1st and the 99th percentile and for each ventile; the 80th and 95th percentiles indicate risk and presence of nomophobia, respectively. Females scored highest across age groups, while older adults reported the lowest levels. These findings support the NMP-Q’s reliability and use in the Italian context.

## 1. Introduction

The evolution of information and communication technologies (ICTs) has reshaped how individuals communicate and access information. This shift has amplified the dynamics of the ‘digital age’ in everyday life (Aceto et al., 2018; Hamelink, 1997; Pérez-Wiesner et al., 2025). However, the pervasive use of smartphones for communication, information retrieval, and social interaction has led to increased reliance on these devices, with psychological consequences when they become unavailable (see Jahrami et al., 2021, 2022). This technological revolution has also contributed to the emergence of new phobias and threats for mental health (Hodkinson, 2019; A. L. S. King et al., 2013). Among these, a particular condition has gained increasing attention, namely, “NO-mobile-phone-phobia,” or nomophobia, which describes the anxiety and fear experienced when individuals cannot use their technological devices for communication (Bhattacharya et al., 2019; Bragazzi & Del Puente, 2014; Gonçalves et al., 2020). On a practical level, nomophobia has been linked to several ‘typical’ symptoms, including anxiety or panic when the phone is misplaced, runs out of battery, or has no network coverage (AlMarzooqi et al., 2022; Farchakh et al., 2021). Individuals may experience obsessive thoughts about checking their phone and staying connected, leading to restlessness or irritability if unable to do so. Physical symptoms can include sweating, trembling, and a rapid heartbeat (Bulut & Sengul, 2024). This condition can also affect daily activities, causing distraction, reduced productivity, and negative impacts on social interactions and mental health (Aguilera-Manrique et al., 2018). Among its typical psychological expressions and correlates, nomophobia has been demonstrated to diminish attentional span, impair working memory, and diminish cognitive performance during complex tasks that require concentration, such as problem-solving or project management (Gezgin et al., 2018; A. L. S. King et al., 2010).

Additionally, it has been linked to increased anxiety, an inclination toward procrastination, and a reduction in the capacity for critical and creative thinking (Cheever et al., 2014). As smartphones have become integral to daily life, this phenomenon has become increasingly prevalent and related by previous studies with a so-called ‘sense of urgency’ (Useche et al., 2024). Furthermore, the prevalence and severity of nomophobia have been growingly associated with demographic factors, especially gender and age (e.g., Kaviani et al., 2020).

### 1.1. Gendered and Age-Based Differences: Nomophobia and Socio-Demographic Factors

Previous studies show that the prevalence and severity of nomophobia are not uniformly distributed. In fact, it varies between 6% and 73% across different populations, with significant associations with demographic factors, particularly gender and age (A. C. León-Mejía et al., 2021 for prevalence variability). Specific studies further suggest that these factors are key in shaping an individual’s reliance on mobile devices and their susceptibility to anxiety when deprived of mobile connectivity (A. C. León-Mejía et al., 2021).

In other words, gender differences are clear across the literature, with research indicating that women may experience higher levels of nomophobia than men, potentially due to different social and communication needs (Arpaci, 2022; Arpaci et al., 2017). Regarding these gender differences, women often use smartphones for social communication, increasing their dependence on these devices and their anxiety when they are unavailable (Bian & Leung, 2015). Furthermore, women are typically more sensitive to social and emotional cues, which can heighten the distress related to being out of touch through mobile communication (Arpaci, 2022). In contrast, men tend to use smartphones for more utilitarian purposes, such as information retrieval and gaming, leading to a different dependency pattern (A. C. León-Mejía et al., 2021; Notara et al., 2021). Additionally, cultural and social expectations related to gender roles may contribute to these differences. Women are often expected to maintain close social and emotional connections, which are facilitated through smartphone use (Walker et al., 2015). Consequently, the disruption of this ‘communication network’ can lead to greater psychological discomfort.

Moreover, as suggested by the limited available literature, age appears to be a clear differentiating factor in nomophobia. Younger individuals, particularly adolescents and young adults, are generally more susceptible, often reporting higher scores and experiencing greater discomfort when disconnected (Gurbuz & Ozkan, 2020). This vulnerability is attributed to their extensive use of smartphones for social networking, academic activities, and entertainment (Moreno-Guerrero et al., 2020). The pervasive integration of mobile technology into their everyday lives fosters strong dependence, increasing their anxiety when disconnected. In contrast, middle-aged and older adults tend to show lower levels of nomophobia, which is related to less frequent use of technology compared to younger individuals. In addition, previous studies have systematically shown how older adults often have difficulty interacting with technological devices in several spheres, compared to younger generations (Lijarcio et al., 2019; Vaportzis et al., 2017). However, it is noteworthy that the gap is narrowing as mobile technology becomes more ubiquitous and integral across all age groups (Fox & Connolly, 2018).

### 1.2. Assessment of Nomophobia: Present and Future Tools and Practices

In recent years, various instruments have been developed to assess the problematic use of technology and nomophobia. Particularly, the Mobile Phone Involvement Questionnaire (MPI-Q) (Walsh et al., 2010) and the Problematic Mobile Phone Use Questionnaire (PMPU-Q) (Billieux et al., 2008) have gained prominence as accurate and valid questionnaires in this pursuit. Overall, these scales are designed to assess different aspects of smartphone usage and potential dependency.

The MPI-Q assesses the degree of an individual’s involvement with their smartphones, covering dimensions such as frequency of use, emotional attachment, and the extent to which phone use interferes with daily activities. It helps identify users who may display problematic behaviors (Walsh et al., 2010).

In contrast, the PMPU-Q focuses on the problematic aspects of smartphone use, including addiction symptoms such as withdrawal, tolerance, and the negative consequences of phone use on daily life (Billieux et al., 2008). It provides a broader view of smartphone dependency and its adverse effects. Both questionnaires offer useful information about smartphone use and dependency. However, they lack specific items addressing the emotional and psychological responses linked to smartphone absence, which is crucial for screening possible cases of nomophobia. At a practical level, and given the rising prevalence of nomophobia, there has been a clear need to identify and develop tools to assess this condition. Yildirim and Correia (2015) proposed a new psychometric tool, the Nomophobia Questionnaire (NMP-Q), for the identification of the levels of nomophobia. The NMP-Q comprises 20 items and four sub-scales that investigate four nomophobia dimensions: “not being able to access information”, “giving up convenience”, “not being able to communicate”, and “losing connectedness”. To date, the NMP-Q has been translated and validated in different countries, including China (Gao et al., 2020), Spain (Gutiérrez-Puertas et al., 2019), Portugal (Galhardo et al., 2020), Mexico (Caba-Machado et al., 2024), and Italy (Adawi et al., 2018). In each of these validations, the original four-factor structure was retained, with the exception of the Italian validation, which proposed a three-factor structure as more appropriate. The psychometric properties of the Italian version of the NMP-Q (Adawi et al., 2018), particularly its factor structure, remain open to debate. This version suggested a three-factor model (Adawi et al., 2018), which may not fully cover the complexity of nomophobia compared to Yildirim and Correia (2015). Moreover, Adawi et al. (2018) did not conduct confirmatory factor analysis (CFA) procedures to assess the fit of the factorial model with the empirical data, nor did they establish normative data population-based for identifying different levels of nomophobia.

### 1.3. The Present Study

Given these points, we are interested in providing further input to the validation of the tool, including the replicability of the proposed results and models. Therefore, the aim of this study is twofold. The first aim is to examine which of the two models (3 or 4 factors) fits our data better. The second aim seeks to establish empirically supported cut-off points for identifying different levels of nomophobia in the Italian population.

Regarding the first aim, several studies have introduced a second-order factor to explain the four dimensions of nomophobia (Caba-Machado et al., 2024; Galhardo et al., 2020; A. León-Mejía et al., 2021 for the methodological approach to determining cut-off points). However, the relationships between each indicator and the higher-order factors are indirect, mediated by first-order factors. This assumption is somewhat rigid and often lacks empirical support (Gignac & Kretzschmar, 2017). Consequently, several studies advocate for the use of bifactorial exploratory structural equation modeling (B-ESEM) as a more effective approach to evaluating the factorial structure of multidimensional constructs (Marsh et al., 2014; Morin et al., 2016).

This method separates the total item covariance into a global component (the G factor), which accounts for the variance shared across all items, and specific factors (S factors) that explain the covariance within subsets of items not captured by the global component. It directly explains the relationship between each indicator (specific factor S) and the higher-order factors (general factor G) (Valenti et al., 2024). Interestingly, the literature has demonstrated that the B-ESEM approach most effectively represents the complexity of the nomophobia construct, yielding optimal fit indices (Valenti et al., 2024).

Regarding the second aim, the results of this validation study are expected to provide researchers and clinicians with a reliable tool for measuring nomophobia in the Italian context. In other words, it is expected that a more accurate factor structure will improve the identification of individuals at risk of nomophobia and inform targeted interventions.

## 2. Materials and Methods

### 2.1. Study Setting

This study employed a cross-sectional, questionnaire-based design. Data collection followed a convenience (pseudo-probabilistic) sampling approach, focusing on the general population. Participants were screened to ensure they were in good physical and psychological health, using a set of inclusion/exclusion criteria prior to participation. A group of supervised trainees conducted brief neuropsychological assessments, gathering demographic information, excluding individuals with neurodegenerative, vision, or hearing disorders (three participants were excluded from the study), and collecting details about participants’ smartphone usage and technological habits. Data were collected between October and December 2023 as part of larger research project at the University of Bari (Italy), focused on studying driving distractions, including the use of technological devices by drivers. The participants were recruited using a multicenter approach involving universities and community partners in the Apulia region in southern Italy. The research team’s institutional affiliations and logistical limitations led to this geographic focus. However, participants were selected from both rural and urban areas in the region, including Bari. Moreover, to maximize intra-regional variability and improve the findings’ internal validity, the sampling strategy included people from a range of age groups and educational backgrounds and targeted both urban and rural populations.

The NMP-Q used in this study was the Italian translation by Adawi et al. (2018). The scale items were translated using the “backward and forward” method, following the steps outlined in both the specialized literature and previous adaptations of scales addressing similar topics (Adawi et al., 2018; Peters & Passchier, 2006).

### 2.2. Ethical Issues

We recognize the significance of a more comprehensive ethical analysis in addition to the official ethics committee approval and the implementation of common safeguards (such as anonymity and informed consent). According to Petousi and Sifaki (2020), identifying possible contextual harm, like over-pathologizing commonplace behaviors or fostering digital stigma, is another aspect of ethical integrity in research. Therefore, to reinforce a preventive and awareness-raising framework, our study was designed to minimize interpretive bias, respect participants’ autonomy, and avoid any labeling or diagnostic implications. Indeed, the research was conducted following the principles set out in the Declaration of Helsinki and approved by the local ethics committees (ET-23-23). Finally, informed consent was obtained from all patients to participate in the study.

### 2.3. Study Sample

A total of 1147 participants took part in the study, including 658 females (age: M ± SD = 40.40 ± 19.30) and 489 males (age: M ± SD = 46.95 ± 19.02), with ages ranging from 18 to 85 years. The sample was designed to cover the entire lifespan to enhance the reliability and representativeness of the study findings. Given its size and diversity, the sample coverage was considered satisfactory, with a N/k ratio adequate (number of participants/number of items = 1147/20 = 57.35) (Kline, 2023) for conducting factorial analysis. The participants of the study were exclusively Italian and white. Information regarding the educational level and other demographic characteristics of the study sample is described in detail in Table 1.

### 2.4. Statistical Analysis

To assess the factorial validity of the Italian version of the NMP-Q, exploratory structural equation modeling (ESEM) was conducted using R Studio (version 4.4.1, 2024). This method was employed to identify the underlying relationships among the measured variables.

The model was evaluated using several goodness-of-fit criteria: chi-square value (χ^2^), comparative fit index (CFI), Tucker–Lewis Index (TLI), root mean square error of approximation (RMSEA), and standardized root mean square residual (SRMR). Different ESEM models were tested to confirm the optimal factorial configuration for the instrument. The thresholds for CFI and TLI were set at >0.900, SRMR and RMSEA at <0.080. In addition, two cut-off points were established to categorize different levels of nomophobia across age categories and divided by gender.

For comparative analyses, analysis of variance (ANOVA) was used to assess age differences, and *t*-tests for independent samples were conducted to examine gender differences, if any. Internal consistencies were assessed by calculating Cronbach’s alpha and McDonald’s omega coefficients for the overall scale (approached as an unifactorial construct) and each subscale, following the literature-based conceivable composition of the scale. Finally, measurement invariance was assessed by gender (male vs. female) and age categories (young adults vs. adults; adults vs. older adults).

## 3. Results

### 3.1. Results of Comparison of Models with the ESEM Approach

Overall, the ESEM outcomes confirm the suitability of a four-factor configuration. The four factors identified are the same as those of the first validation: (1) Not being able to communicate; (2) Losing connectedness; (3) Not being able to access information; (4) Giving up convenience (Yildirim & Correia, 2015). Factor 1 explains the 17.9% of item variance. Factor 2 explains the 14.9% of item variance. Factor 3 explains the 11.8% of item variance. Factor 4 explains the 10.0% of item variance. To further examine the factorability of the matrix, Bartlett’s test of sphericity was employed to analyze both partial and bivariate correlations. Additionally, the Kaiser–Meyer–Olkin (KMO) measure was used to evaluate sampling adequacy. Bartlett’s test of sphericity yielded significant results (χ^2^ = 17,831, df = 190; *p* < 0.01), rejecting the null hypothesis that the correlation matrix was an identity matrix with zero off-diagonal correlations (Williams et al., 2010). Regarding sampling adequacy, the KMO index was 0.955. Consequently, these findings confirmed that factor analysis was adequate for the data (Kaiser & Rice, 1974).

In order to examine different possible compositions of the NMP-Q, different ESEM models were tested. We evaluated one factor, combinations of two and three factors from different factor solutions, and four factors to test the EFA results.

Additionally, we evaluated the bifactor model with a four-factor solution (B-ESEM). We chose to test this factorial configuration because, in the B-ESEM solution, the associations between general and specific factors are direct. This means that all indicators load onto both a general factor (Factor G) and specific factors (Factor S). The variance of the items is divided into three sources: (a) the specific factor, (b) the general factor, and (c) measurement error. For these reasons, it has been suggested that unless there is a strong theoretical justification for a higher-order representation, bifactor models should be preferred (Marsh et al., 2014; Valenti et al., 2024).

**Figure 1:**
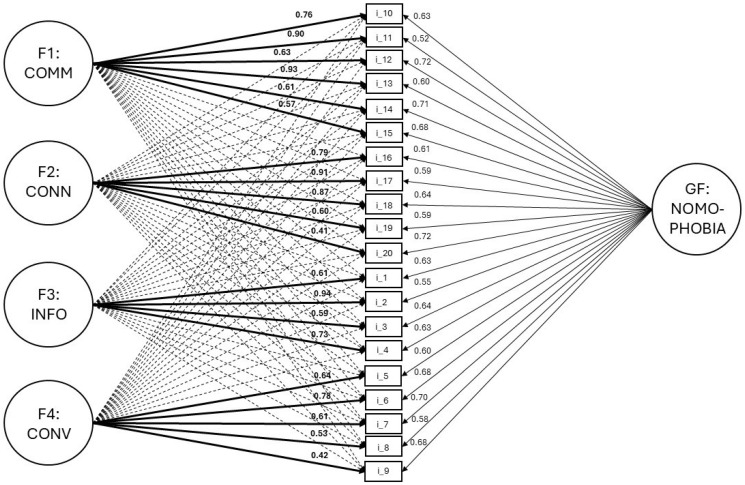
Bifactor exploratory structural equation modeling with four factors (B-ESEM) with standard estimates. Notes: GF: NOMOPHOBIA = general factor of nomophobia; F1: COMM = not being able to communicate; F2: CONN = losing connectedness; F3: INFO = not being able to access information; F4: CONV = giving up convenience.

The main results of comparisons between models are shown in Table 2. The best-fitting model was Model 1, which featured the anticipated B-ESEM solution. Considering the ESEM findings and the indices, the B-ESEM model with four-factor results in the best factorial configuration, with CFI, TLI, SRMR, and RMSEA being satisfactory (CFI: 0.960; TLI: 0.940; SRMR: 0.03; RMSEA: 0.060; Byrne, 2001). Finally, the standardized estimations for all the parameters are significant (<0.001). The comparison between the models makes it clear that the others factorial configurations are not adequate. Indeed, the chi-square difference tends to increase from Model 1 (Δχ^2^ = 426; *p* < 0.001) to Model 13 (Δχ^2^ = 3463; *p* < 0.001). Furthermore, alternative models failed to adequately fit the data, rendering these solutions unacceptable. Therefore, the conclusion is that the four-factor solution using items in the Italian language is the optimal choice, aligning well with the original factorial configuration (Yildirim & Correia, 2015). The bifactor exploratory structural equation model with four factors (B-ESEM) plot with standard estimates (λ) is shown in Figure 1.

### 3.2. Reliability and Internal Consistency

The four-factor configuration of the NMP-Q shows good internal consistency, with Cronbach’s and McDonald’s alpha coefficients exceeding 0.800 for each subscale and for the overall scale (α/ω ≈ [0.850–0.930]; overall α/ω ≈ 0.950), suggesting an adequate level of internal consistency for the questionnaire appraised both from an unifactorial perspective and from its four specific and psychometrically endorsed subscales.

Table 3 presents Cronbach’s alpha and McDonald’s omega values if an item is deleted. Based on the results, each item can be considered indispensable, as removing any item from a NMP-Q subscale either decreases or maintains the Cronbach’s alpha and McDonald’s omega values, according to the four-factor composition shown in Figure 1.

### 3.3. Cut-Off Points of the Italian Version of NMP-Q for Age and Gender

Cut-off points for different age categories were established: young adults (18–29 years old), adults (30–64 years old), and older adults (65+ years old). The mean and standard deviation of the NMP-Q for females was 68.8 (SD = 25.2), while for males it was 59.7 (SD = 24.9). We have identified significant differences between age and gender in the total score of the NMP-Q. The sex differences have been observed through a *t*-test for independent samples (*t* = 6.13; df = 1145; Cohen’s *d* = 0.366; *p* < 0.001), while the age differences have been observed in all three age categories using a one-way ANOVA (*F* = 59.5; *p* < 0.001).

Moreover, the post-hoc tests showed significant differences between young adults and adults (Mean difference = −14.8; *p* < 0.001), young adults and older adults (Mean difference = 25.7; *p* < 0.001), and adults and older adults (Mean difference = 10.9; *p* < 0.001). Two cut-off points have been identified, corresponding to the 80th and 95th percentiles for males and females and for each of the three age categories. The two percentiles correspond to the following categories risk of nomophobia and nomophobia, respectively.

Such cut-off points have been established following the existing literature on the topic. Indeed, the A. León-Mejía et al. (2021) study and the González-Cabrera et al. (2017) study have identified these cut-off points in line with other technological contexts, such as gaming disorders, pathological gambling, and problematic use of the technology. In light of this, we have adapted the nomophobia construct to these categories, as reported by A. León-Mejía et al. (2021) and González-Cabrera et al. (2017). Table 4 presents the percentile scores for each age category, split by gender.

### 3.4. Multi-Group Gender and Age Invariances

Multi-group invariance analysis was conducted on subgroups defined by gender and age categories. The configural model demonstrated a good fit to the data (S-Bχ^2^ = 1768.938, df = 328, RMSEA = 0.055, CFI = 0.916), suggesting that the structure of the instrument was similar across both gender groups.

Next, metric invariance was assessed. The results showed that the metric invariance model fit the data well (S-Bχ^2^ = 1776.627; df = 344, RMSEA = 0.062; CFI = 0.916). The non-significant change in χ^2^ (Δχ^2^ = 7.689; *p* > 0.050) and negligible changes in CFI (ΔCFI = 0.001) and RMSEA (ΔRMSEA = 0.001) suggest that factor loadings were largely equivalent across gender.

Moreover, scalar invariance (a more restrictive level, assessing the equivalence of intercepts) was also examined. While the scalar invariance model showed an acceptable fit (S-Bχ^2^ = 1829.497; df = 364; RMSEA = 0.080; CFI = 0.912), the significant change in χ^2^ (Δχ^2^ = 52.870; *p* < 0.001) and the changes in CFI (ΔCFI = 0.010) and RMSEA (ΔRMSEA = 0.011) suggest that the scalar invariance was not supported.

A similar procedure was followed to investigate variations by age categories. The configural model, testing for equivalence in the factorial structure’s form, demonstrated acceptable fit (S-Bχ^2^(492) = 1437.301; *p* < 0.001; RMSEA = 0.070; CFI = 0.912), suggesting that the same latent factors underlie the observed variables in both groups, namely young adults and adults. The metric invariance model also showed an acceptable fit (S-Bχ^2^(524) = 1468.825; *p* < 0.001; RMSEA = 0.075; CFI = 0.910). The non-significant change in χ^2^ (Δχ^2^(32) = 31.524; *p* = 0.69) and minimal changes in CFI (ΔCFI = 0.003) and RMSEA (ΔRMSEA = 0.005) indicate that the relationships between the items and their respective factors were largely equivalent across young adults and adults. The scalar invariance model, however, showed a statistically significant change in χ^2^ (Δχ^2^(40) = 156.926; *p* < 0.001), suggesting a lack of scalar invariance. Also, the changes in CFI (ΔCFI = 0.013) and RMSEA (ΔRMSEA = 0.009) indicate that the scalar invariance was not supported.

Finally, the configural model exhibited acceptable fit for adults and older adults (S-Bχ^2^(328) = 1347.900; *p* < 0.001; RMSEA = 0.078; CFI = 0.924). The metric invariance model also showed a considerably acceptable fit (S-Bχ^2^(343) = 1380.758; *p* < 0.001; RMSEA = 0.080; CFI = 0.911), as shown in detail in Table 5. While the change in χ^2^ was statistically significant (Δχ^2^(15) = 32.841; *p* < 0.010), the small changes in CFI (ΔCFI = 0.006) and RMSEA (ΔRMSEA = 0.002) suggest that factor loadings remained equivalent across these age groups. However, the scalar invariance model revealed a statistically significant change in χ^2^ (Δχ^2^(17) = 38.584; *p* < 0.010), indicating a lack of scalar invariance. Also, as in the previous case, the changes in CFI (ΔCFI = 0.009) and RMSEA (ΔRMSEA = 0.006) indicate that the scalar invariance was not supported.

## 4. Discussion

The aims this study was to validate the Italian version of the NMP-Q and shed light on nomophobia measurement. From a psychometric perspective, we examined the NMP-Q, offering important information on its factorial structure, reliability, and population-based cut-off points, as a self-report tool under optimal methodological assumptions.

The following section discusses these key aspects in light of previous research, relevant theoretical frameworks, psychometric results, descriptive findings, and practical implications from the present study.

### 4.1. Three or Four? How Does the NMP-Q Fit the Best?

The first aim of this study was to evaluate the psychometric properties of the NMP-Q and to determine whether a four-factor model offers a more accurate representation of the nomophobia construct with respect to a previously validated three-factor model for the Italian population. The results of the B-ESEM support the superiority of the four-factor model, consistent with that concluded by (Valenti et al., 2024). Particularly, the B-ESEM model with four factors, including a general factor (G) and four specific factors (S), demonstrated a significantly better fit than alternative models.

From a psychometric approach, this is supported by the ordinal and error-related fit indices obtained in the retained model, where both the CFI and TLI exceeded the threshold of 0.900, and the RMSEA was below 0.080 (Hu & Bentler, 1999). These indices suggest that the four-factor B-ESEM model aligns well with the data, consistent with the theoretical framework proposed by Yildirim and Correia (2015). Furthermore, chi-square tests reinforced the inadequacy of alternative factorial configurations, further substantiating the robustness of the four-factor solution (see A. C. León-Mejía et al., 2021).

Considering previous research, this factorial structure appears to be more aligned with a four-factor version of the NMP-Q globally. For example, the Chinese validation of the instrument supports a four-factor model (Gao et al., 2020), as does the Mexican validation, which includes the same factors identified in Yildirim and Correia’s original model and incorporates a second-order factor explaining the relationships among the four dimensions of nomophobia (Caba-Machado et al., 2024).

The same theoretical structure has been found in the Spanish and Portuguese validations (Galhardo et al., 2020; A. C. León-Mejía et al., 2021). Upon examination of both these studies and the present outcomes, there are converging evidences that a four-factor configuration provides a more comprehensive representation of the multidimensional nature as well as of the psychological correlates and expressions of nomophobia. We adopted the methodological approach proposed by Valenti et al. (2024) and employed a B-ESEM model. This method captures the relationships between the specific factors (S) and the higher-order general factor (G). It improves the structural clarity of the NMP-Q and strengthens the validity of the four-factor model compared to the three-factor model.

### 4.2. Gender and Age-Based Cut-Off Scores: Examining Nomophobia Variability

Regarding the second aim of this study, we identified different cut-off scores for males and females. Overall, these thresholds differ from those proposed by A. León-Mejía et al. (2021) and González-Cabrera et al. (2017) but follow a similar pattern. Additionally, our results indicate that females consistently report higher levels of nomophobia than males across all age categories, a trend widely documented in the literature, which often highlights greater technology affinity and related concerns among female individuals (Gezgin et al., 2018; O’Hern et al., 2025).

Regarding age differences, these findings align with previous findings suggesting that younger individuals are more susceptible to nomophobia (Dasgupta et al., 2017), with levels peaking during adolescence and early adulthood (King et al., 2017). Conversely, the cut-off points for older adults were considerably lower than those observed in younger groups, reinforcing the literature-supported idea that nomophobia and problematic technology use tend to be less prevalent among older individuals (Gurbuz & Ozkan, 2020).

In addition to identifying cut-off points, the internal consistency of the NMP-Q was evaluated using Cronbach’s alpha and McDonald’s omega, (R studio, 4.4.1) which were adopted as complementarily reliably indicators of the scale’s internal consistency. Looking at the numbers, both unifactorial coefficients approached 0.950, while subscale alphas ranged between 0.850 and 0.930, all exceeding conventional reliability thresholds. In other words, these results indicate a strong internal consistency, confirming that the items within each subscale effectively measure their respective constructs. The robustness of these reliability indices further supports the validity of the four-factor structure and highlights the NMP-Q’s suitability for assessing nomophobia across different demographic groups.

While the current study provides strong evidence for the validity and reliability of the 4-factor model, it is not without limitations. First of all, the social desirability bias may influence the participants’ responses. However, this bias is mitigated in part by the anonymity of responses in our study. Moreover, the use of a convenience sample may limit the generalizability of the findings. Future research should aim to replicate these results in more diverse and representative samples to improve the generalizability of the findings, including adolescents (A. C. León-Mejía et al., 2021; González-Cabrera et al., 2017). Additionally, longitudinal studies are needed to examine the stability of the factor structure over time and to explore the causal relationships between nomophobia and its psychological and behavioral correlates. Finally, full scalar invariance was not supported across gender and age groups, although the changes in fit indices remained within acceptable ranges. These results suggest that partial invariance may allow for cautious comparisons of latent means across groups. The practical implications of this phenomenon require further elucidation. Minor deviations from scalar invariance indicate the possibility of items functioning differently across groups. However, the B-ESEM model’s robustness and the subscales’ substantial internal consistency substantiate the validity of mean score comparisons.

Finally, it is important to highlight that the study’s cut-off scores are helpful indicators for determining who is more statistically likely to experience nomophobia, but they should not be used as a guide for clinical diagnosis. The reported percentiles represent normative statistical distributions rather than clinical standards and are derived from a particular regional sample. As a result, care should be taken when using these cut-offs in different clinical or cultural contexts, and additional research is required before concluding their clinical significance. Their main aim is to assist comparative research and early detection, not to take the place of clinical evaluation.

## 5. Conclusions

This study proposed a validation of the four-factor structure of the No-Mobile-Phone-Phobia Questionnaire in Italian population, confirming its reliability and utility as a self-report tool for assessing nomophobia. Its core conclusions, raised in accordance with the study aims and their practical implications, are summarized below.

First, the findings of this research support the psychometric suitability of the NMP-Q, as well as its applicability in research screening contexts, facilitating the identification of individuals at higher risk and informing targeted interventions.

Second, this study identifies sensitive gender and age differences in nomophobia levels. Females consistently report higher nomophobia scores across age groups, while younger individuals exhibit greater susceptibility compared to older adults. These patterns reinforce the need for age- and gender-sensitive approaches in future research and intervention strategies.

On a practical level, the robust metric properties of the Italian NMP-Q make it a valuable instrument for investigating nomophobia in both academic and applied settings. Moreover, the alignment of the Italian version with the original factorial structure, composed of four literature-coherent dimensions, enhances its potential for cross-cultural comparisons following the same theoretical approach.

In summary, this study has adapted a new factorial configuration with superior fit and robust psychometric properties. Nevertheless, future research should focus on more diverse and representative samples, including adolescents, and explore the stability of the factorial structure over time.

## Figures and Tables

**Table 1:** Descriptive statistics.

					Skewness		Kurtosis	
	Gender	N	M	SD	Skewness	S.E.	Kurtosis	S.E.
NMP-Q	F	658	68.83	25.18	0.118	0.095	−0.509	0.190
	M	489	59.66	24.89	0.371	0.110	−0.357	0.220
AGE	F	658	40.38	19.26	0.366	0.095	−0.206	0.190
	M	489	46.95	19.01	0.002	0.110	−0.258	0.220
EDU	F	657	13.58	3.96	−0.372	0.095	0.322	0.190
	M	488	12.56	4.04	0.103	0.110	0.568	0.221

**Table 2:** Results of ESEM comparison models (Model 13 to Model 1).

Model Type	MODEL	χ^2^	df	*p*	CFI	TLI	SRMR	RMSEA	Model Comparison	Δχ^2^	df	*p*
M13	1-factor configuration	5001	170	0.001	0.72	0.68	0.09	0.15	M13-M1	3463	6	0.001
M12	2 factors (1 + 2), (3 + 4)	4355	169	0.001	0.75	0.72	0.09	0.14	M12-M1	2817	5	0.001
M11	2 factors (1 + 3), (2 + 4)	4161	169	0.001	0.76	0.74	0.09	0.14	M11-M1	2624	5	0.001
M10	2 factors (1 + 4), (2 + 3)	3494	169	0.001	0.80	0.78	0.09	0.13	M10-M1	1956	5	0.001
M9	3 factors (3 + 4), 2, 1	3812	167	0.001	0.79	0.76	0.08	0.13	M9-M1	2274	3	0.001
M8	3 factors (1 + 2), 3, 4	3812	167	0.001	0.79	0.76	0.08	0.13	M8-M1	2274	3	0.001
M7	3 factors (1 + 3), 2, 4	3049	167	0.001	0.83	0.81	0.09	0.12	M7-M1	1511	3	0.001
M6	3 factors (2 + 3), 1, 4	2825	167	0.001	0.84	0.82	0.07	0.11	M6-M1	1288	3	0.001
M5	3 factors (2 + 4), 1, 3	2662	167	0.001	0.85	0.83	0.07	0.11	M5-M1	1124	3	0.001
M4	3 factors (1 + 4), 2, 3	2231	167	0.001	0.88	0.86	0.07	0.10	M4-M1	694	3	0.001
M3	4 factors (1 + 2 + 3 + 4)	1538	164	0.001	0.92	0.91	0.06	0.08	M3-M1	604	24	0.001
M2	Bifactor model with 3 factors	1261	144	0.001	0.93	0.92	0.06	0.08	M2-M1	426	4	0.001
M1	Bifactor model with 4 factors	835	140	0.001	0.96	0.94	0.03	0.06				

Notes: χ^2^ = chi-square; df = degrees of freedom; CFI = confirmative fit index; TLI = Tucker–Lewis Index; SRMR = standardized root mean square residual; RMSEA = root mean square error of approximation; Δχ^2^ = delta chi-square; *p* = *p*-value.

**Table 3:** Results of ESEM and reliability analyses of the full scale and its subscales.

Items	λ	Cronbach’s Alpha If Item Deleted	McDonald’s Omega If Item Deleted	Cronbach’s Alpha	McDonald’s Omega
Factor 1 (Not being able to communicate)				0.932	0.931
13	0.934	0.911	0.912		
11	0.902	0.919	0.919		
10	0.767	0.916	0.917		
12	0.630	0.919	0.919		
14	0.616	0.921	0.922		
15	0.567	0.930	0.930		
Factor 2 (Losing connectedness)				0.895	0.902
17	0.913	0.854	0.861		
18	0.876	0.849	0.859		
16	0.799	0.865	0.875		
19	0.601	0.892	0.901		
20	0.410	0.895	0.902		
Factor 3 (Not being able to access information)				0.879	0.880
2	0.941	0.824	0.826		
4	0.731	0.840	0.841		
1	0.605	0.857	0.859		
3	0.590	0.824	0.860		
Factor 4 (Giving up convenience)				0.853	0.855
6	0.784	0.817	0.817		
5	0.642	0.818	0.822		
7	0.615	0.815	0.817		
8	0.532	0.828	0.831		
9	0.470	0.838	0.840		
Full scale (Unifactorial composition)				0.951	0.952

Notes: λ = Lambda standardized estimates of factor loadings.

**Table 4:** Cut-off scores split by age and gender.

		Male			Female	
Percentile	Young Adults (18–29) *n* = 140	Adults (30–64) *n* = 248	Older Adults (65–85) *n* = 102	Young Adults (18–29) *n* = 280	Adults (30–64) *n* = 287	Older Adults (65–85) *n* = 92
1th	20	20	20	29	20	20
5th	33	25	20	42	28	20
10th	40	29	20	49	36	20
15th	43	33	20	55	40	21
20th	47	36	20	59	43	25
25th	52	40	25	65	48	29
30th	55	43	28	69	51	33
35th	59	46	34	72	53	38
40th	61	49	39	74	56	40
45th	65	53	42	76	60	41
50th	69	57	46	79	62	43
55th	71	61	50	83	66	46
60th	74	63	54	87	68	53
65th	78	66	59	90	71	60
70th	79	72	65	93	74	65
75th	81	75	67	96	78	73
**80th**	**87**	**79**	**71**	**100**	**82**	**76**
85th	92	83	79	106	89	80
90th	95	89	89	110	93	83
**95th**	**108**	**102**	**96**	**120**	**105**	**97**
99th	127	119	100	135	118	97
M	68.0	58.5	49.1	80.1	63.7	50.2
SD	22.3	9.6	24.8	22.8	22.3	24.1

Notes: Bold rows indicate the 80th and 95th percentiles.

**Table 5:** Multi-group gender and age invariances of NMP-Q scores.

Model	df	S-Bχ^2^	RMSEA	CFI	Diff. χ^2^	∆CFI
Gender						
Configural Invariance	328	1768.938	0.055	0.916	-	-
Metric Invariance	344	1776.627	0.062	0.916	7.689	0.000
Scalar Invariance	364	1829.497	0.080	0.912	52.870 ***	0.005
Age						
Young Adults vs. Adults						
Configural Invariance	492	1437.301	0.070	0.912	-	-
Metric Invariance	524	1468.825	0.075	0.910	31.524	0.003
Scalar Invariance	564	1624.332	0.079	0.901	156.926 ***	0.011
Adults vs. Older Adults						
Configural Invariance	328	1347.900	0.078	0.924	-	-
Metric Invariance	343	1380.758	0.080	0.911	32.841 **	0.009
Scalar Invariance	360	1419.302	0.086	0.903	38.584 **	0.009

Notes: S-Bχ^2^ = Satorra–Bentler chi-square; df = degrees of freedom; RMSEA = root mean square error of approximation; CFI: Comparative fit index; Diff. χ^2^ = chi-square difference; ∆CFI = differences in the comparative fit index; ** *p* < 0.010; *** *p* < 0.001.

## Data Availability

The raw data supporting the conclusions of this article will be made available by the authors upon reasonable request.

## Abbreviations

The following abbreviations are used in this manuscript:

NMP-Q	No-Mobile-Phone-Phobia-Questionnaire
ESEM	Exploratory Structural Equation Modeling
